# Probing the Spatial Organization of Molecular Complexes Using Triple-Pair-Correlation

**DOI:** 10.1038/srep30819

**Published:** 2016-08-22

**Authors:** Yandong Yin, Eli Rothenberg

**Affiliations:** 1 Department of Biochemistry and Molecular Pharmacology, New York University School of Medicine, 550 1st Avenue, New York, NY 10016, USA

## Abstract

Super-resolution microscopy coupled with multiplexing techniques can resolve specific spatial arrangements of different components within molecular complexes. However, reliable quantification and analysis of such specific organization is extremely problematic because it is frequently obstructed by random co-localization incidents between crowded molecular species and the intrinsic heterogeneity of molecular complexes. To address this, we present a Triple-Pair-Correlation (TPC) analysis approach for unbiased interpretation of the spatial organization of molecular assemblies in crowded three-color super-resolution (SR) images. We validate this approach using simulated data, as well as SR images of DNA replication foci in human cells. This demonstrates the applicability of TPC in deciphering the specific spatial organization of molecular complexes hidden in dense multi-color super-resolution images.

Optical super-resolution (SR) imaging techniques greatly surpass diffraction-limited microscopy, enabling the visualization of subcellular architectures with an accuracy of tens of nanometers^1^,^2^,^3^,^4^. In particular, when extended to multicolor imaging, SR can potentially resolve individual morphologies, as well as the specific internal spatial arrangement of molecular complexes^5^,^6^,^7^. However, the enhanced detection sensitivity of SR imaging and the resulting highly detailed information imposes serious challenges for accurate and unbiased quantification of the specific features of molecular complexes. This is especially critical for analysis of images containing particularly dense and abundant molecular species that are subjected to heterogeneous distribution, various orientations, and random co-localization incidents^8^,^9^. The recent advent of local-density and temporal-stochastic based segmentation methods^10^,^11^,^12^ has improved the quantification of single-color SR images with high molecular densities. In these methods the geometric center of molecular clusters is identified from their local spatial descriptive statistics, which can then be applied for analysis of Nearest-Neighboring-Distances (NND) between molecules labeled with different colors. However, in the case of multicolor molecular clusters, NND analysis cannot distinguish truly correlated species from randomly colocalized ones, and as a result, non-specific and random co-localization events inevitably dominate the NND distribution. Alternatively, Cross-Pair-Correlation analysis of SR images is capable of recognizing correlated molecules and calculating their correlation distances^13^, but such distance information between two components ultimately cannot be used for quantification of complicated molecular complexes, especially complexes composed of more than two components that are nonlinearly arranged.

Here, we report a Triple-Pair-Correlation (TPC) approach^14^ for unbiased analysis of the organization of molecular complexes in three-color SR images. The TPC approach generates a correlation profile derived from three independent geometric features, providing accurate quantification of the spatial arrangements of three different species labeled with different colors within a specific molecular assembly.

## Results and Discussion

**Figure 1:**
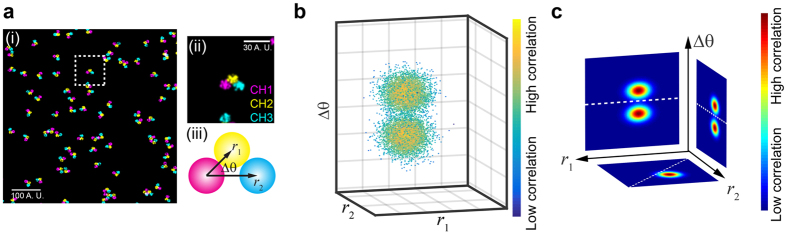
Conceptual illustration of Triple-Pair-Correlation function. (**a**) Simulated molecular complex randomly orientated and localized in a three-color image (i), with each complex composed of three different colored elements (ii) and its internal spatial organization defined by (*r*_1_, *r*_2_, Δ*θ*) (iii). (**b**) Schematic illustration of the 3D Triple-Correlation *g*(*r*_1_, *r*_2_, Δ*θ*) corresponding to the geometric properties shown in (**a**). (**c**) Three 2D correlation maps obtained through one dimensional integration of (**b**).

For simplicity, we annotate the three different color channels as Channels 1–3, as shown in the simulated data in Fig. 1a. The TPC algorithm calculates the probability of simultaneously finding three different molecules, each in a different channel, as a function of their relative displacement. Specifically, for each molecule located at vector coordinates **R** in channel 1 (CH1), the average probability of finding a molecule located at **R + r**_1_ in channel 2 (CH2), and another molecule located at **R + r**_2_ in channel 3 (CH3) is given by the TPC function (equation (1) and Fig. 1a(iii)):


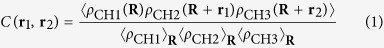


where 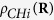 is the molecular density detected at position **R** = (*R*, *θ*) in channel *i*. Since the molecular complexes can be randomly orientated 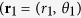 and 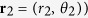, their internal correlation is derived from their relative orientation 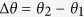 rather than an absolute one (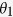 and 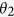). Hence, the TPC only relies on three variables, and is written as: 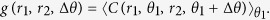 Figure 1b shows an illustrative TPC 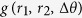 plot of the molecules shown in Fig. 1a, reaching the highest response at a specific combination of 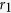, 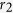, and 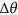 which represents the internal spatial arrangement of the three molecules. Note that the molecular pattern can be imaged as a pair of reflectional symmetries in 2D (facing up and down), and therefore 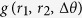 has an equal response in the 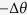 region (Fig. 1b). To generate a more comprehensive mapping in the TPC cube, we integrated 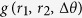 through each dimension, 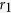, 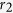, and 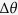, resulting in a combination of three 2D correlation maps (Fig. 1c).

Similar to the Pair Correlation function^15^, the TPC function can be represented as equation (2) (see Supplementary Note 1 for derivation):


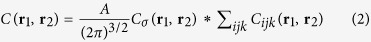


where 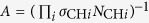 with 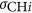 and 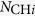 denote the localization uncertainty and number of fluorophores in channel *i*, respectively; the effective point spread function of fluorophores in CH*i* is described as 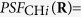

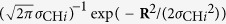
, and 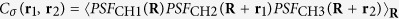 is their triple correlation, representing how localization uncertainties contribute to the whole TPC function; 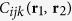 quantifies the spatial correlation among the *i*th fluorophore from CH1, the *j*th fluorophore from CH2, and the *k*th fluorophore from CH3; and *denotes convolution.

**Figure 2:**
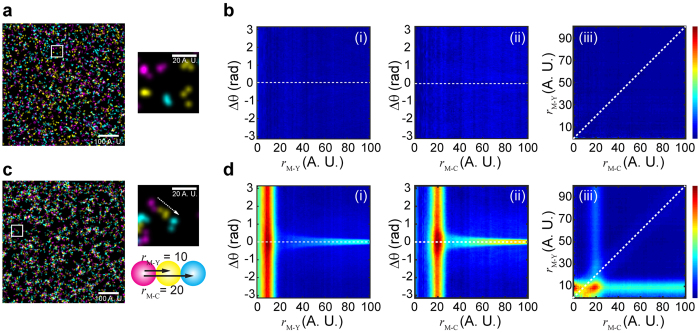
Triple-Pair-Correlation resolves simulated molecular pattern in highly dense SR image. (**a**) Simulated molecules that randomly distribute in all the three channels. (**b**) Triple-Pair-Correlation maps of simulated images of (**a**) displays no spatial correlation between molecules from all the three channels. (**c**) Simulated molecules that linearly arrange into a certain sequential pattern. (**d**) Triple-Pair-Correlation analysis well resolved the internal spatial organization of molecular complex in (**c**) (Supplementary Figure S1). The feature appearing at 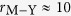, 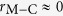 is a residual signal arising from the indirect bispectrum calculation (Supplementary Note 2), and accordingly can be subtracted as such. Color bar from blue to red represents low to high correlation amplitude, respectively. Images shown in (**a**,**c**) were Gaussian blurred for display purpose.

When the fluorophores from different channels randomly distribute relative to each other (Fig. 2a), 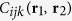 is constantly equal to 1, and consequently the TPC function, as a result of the convolution between 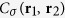 and 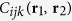, represents a constant (Fig. 2b(i–iii)). On the other hand, when the fluorophores are arranged into certain molecular patterns, the whole TPC function quantifies the internal spatial organization of the molecular patterns as a function of 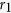, 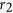, and 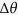 (Fig. 1). Figure 2c shows simulated molecular patterns each composed of three fluorophores of different colors (designated as M, Y, and C), and their TPC maps (Fig. 2d (i–iii)), which display a unique response at 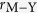 = ~8.3 (A.U.), *r*_M−C_ = ~19 (A.U.), and Δ*θ* = ~0 (deg.) according to a Gaussian fit (Supplementary Figure S1), in good agreement with the pre-assigned geometry (Fig. 2c). We note that the localization uncertainty does not affect the position of the TPC profile within the *g*(*r*_1_, *r*_2_, Δ*θ*) cube (or maps), but that an increasing localization uncertainty will widen the TPC profile (Supplementary Figure S2). Meanwhile, if the fluorophores of the same color accumulate into a cluster, and the clusters of different colors are then arranged into certain patterns, an increasing size of clusters will also widen the TPC profiles (Supplementary Figure S3). We also simulated molecular patterns that have non-linear internal organizations, and their non-zero Δ*θ* are well resolved by the TPC function (Supplementary Figure S4).

**Figure 3:**
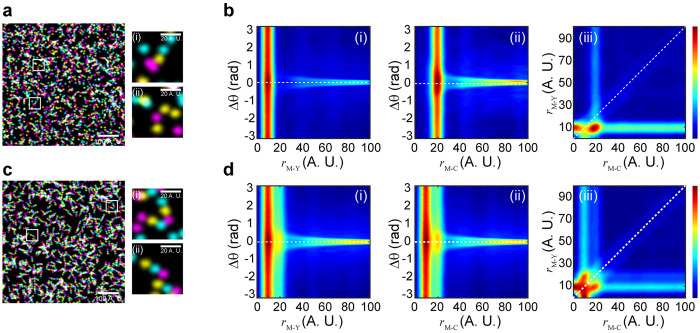
Two scenarios of simulated SR images that contain heterogeneous molecular complex. (**a**) Simulated SR image that contains ~49% spatially arranged molecular complex (i) and ~51% randomly distributed molecules (ii). (**b**) Triple-Pair-Correlation maps of simulated images of (**a**), succeeding in resolving the spatial organization of the molecular complex from randomly distributed molecules. (**c**) Simulated SR image that contains ~50% M-Y-C pattern (i) and ~50% M-C-Y pattern (ii). (**d**) The Triple-Pair-Correlation analysis well resolved the heterogeneity of molecular complex with different internal organization (Supplementary Figure S5). Color bar from blue to red represents low to high correlation amplitude, respectively. Images shown in (**a**,**c**) were Gaussian blurred for display purpose.

To further test if this approach is capable of resolving the heterogeneity of molecular patterns present in the same image, we carried out TPC analysis on simulated images with molecular patterns that have heterogeneous distributions. We first simulated a scenario where only functional molecules can form a specific pattern that is shown in Fig. 2c (Fig. 3a(i)), while non-functional molecules are randomly distributed (Fig. 3a(ii)). Figure 3b(i–iii) shows the TPC analysis of this scenario, where functional molecular patterns can be well distinguished from those randomly distributed molecules. We also tested the TPC approach by analyzing two different patterns present in the same image (Fig. 3c). In this scenario, half of the molecules form a Magenta-Yellow-Cyan (M-Y-C, Fig. 3c(i)) pattern and the other half form a Magenta-Cyan-Yellow (M-C-Y, Fig. 3c(ii)) pattern (i.e. 50% M-Y-C vs. 50% M-C-Y). While the resulting TPC profiles (Fig. 3d(i–iii)) show both populations peaking at *r* = ~9 and *r* = ~19 (A.U.) as a function of either *r*_M−Y_ or *r*_M−C_, the two different patterns are well resolved in Fig. 2f(iii), with two distinct populations peaking at *r*_M−Y_ = ~9 & *r*_M−C_ = ~19 and *r*_M−Y_ = ~20 & *r*_M−C_ = ~9 (A.U.) (Supplementary Figure S5). This demonstrates the advantage of TPC analysis over other methods, especially since the Cross-Pair-Correlation between M and Y and between M and C is the same, so that the two patterns cannot be resolved by a Cross-Pair-Correlation approach.

Next, we tested the TPC approach by analyzing three-color SR imaging data examining DNA replication in human U2OS cells. DNA replication is performed by the replisome, a multifunctional protein machine that orchestrates parental DNA unwinding, single-stranded DNA stabilization, and nascent DNA synthesis. As viewed by diffraction-limited microscopy, replisome proteins form bright replication foci where the different replication factors co-localize with nascent chromatin^16^. Both nascent DNA and replisome proteins are highly abundant in the nucleus during S-phase, resulting in random overlap of the different replisome components^17^. To examine whether the TPC approach can distinguish the arrangements of the active replisomes subpopulation from random species, we carried out three-color imaging of cells where nascent DNA and the replication proteins PCNA and RPA were labeled (Methods). We note that RPA specifically binds to single-stranded DNA at replication forks and at other locations, further increasing the difficulty of resolving and quantifying individual replications units within such data.

**Figure 4:**
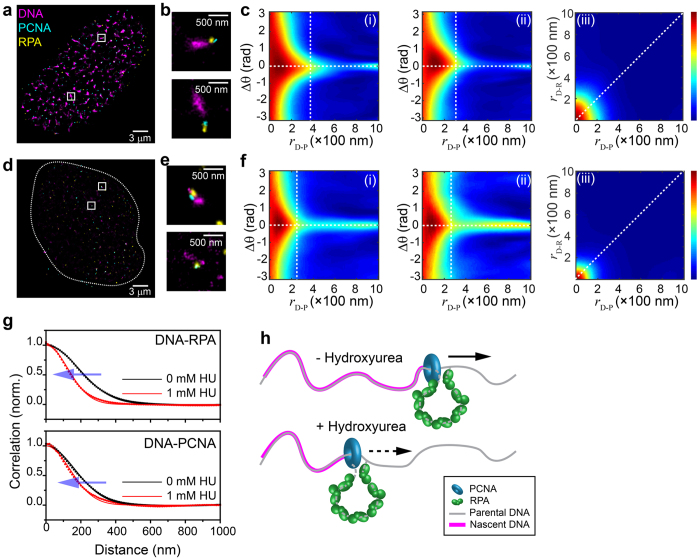
Triple-Pair-Correlation analysis of the SR resolved spatial arrangement of replisomes in cells. (**a**) Mutlicolor SR image of a U2OS cell nucleus in early S-phase with nascent DNA, PCNA, and RPA stained in magenta, cyan, and yellow, respectively. (**b**) Two magnified images showing examples of the internal spatial organization of a replication focus. The experimental localization accuracies for the different colors used in these measurements are: 15 nm, 37 nm, and 44 nm for magenta (Alexa Fluor 647), yellow (Alexa Fluor 568), and cyan (Alexa Fluor 488), respectively (see Data Analysis in Methods section). (**c**) Triple-Pair-Correlation maps obtained by averaging the Triple-Correlation of 13 nuclei. Distances from DNA to RPA (*r*_D−R_) and from DNA to PCNA (*r*_D−P_) are ~149.7 ± 0.6 and 132.0 ± 0.7 nm, respectively; angles in between *r*_D−R_ and *r*_D−P_ follows a Gaussian distribution centering at 0 with standard deviation of ~29 deg. (Supplementary Figure S6). (**d**) Mutlicolor SR image of a U2OS cell nucleus treated with 1 mM HU in early S-phase. (**e**) Two magnified images showing examples of the internal spatial organization of a replication focus under replication stress. (**f**) Triple-Pair-Correlation maps obtained by averaging the Triple-Pair-Correlation of 18 nuclei. Distances from DNA to RPA (*r*_D−R_) and from DNA to PCNA (*r*_D−P_) are ~100 ± 1 and 109 ± 1 nm, respectively. Angles in between *r*_D−R_ and *r*_D−P_ follows a Gaussian distribution centering at 0 with standard deviation of ~20 deg. (Supplementary Figure S8, all errors are propagated fitting errors). (**g**) 1D correlation of *r*_D−R_ (top) and *r*_D−P_ (bottom) obtained via integration of *r*_D−R_ − *r*_D−P_ correlation map (**c**(iii),**f(**iii)) along *r*_D−P_ and *r*_D−R_, respectively. Both graphs display a decrease of correlation distances when cells treated with 1 mM HU. Dots and lines represent the raw data and modified Gaussian fit, respectively (Supplementary Note 3). We note that the overlapped portion between nascent DNA and replisome proteins resulted in a high Triple-Correlation response at *r*_D−R_ (or *r*_D−P_) of ~0 nm covering all 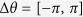, which further resulted in a high correlation at 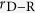 (or 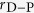) of ~0 nm after integration through 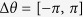 in (**c**(iii),**f**(iii),**g**) (Supplementary Note 3 and Supplementary Figure S7). (**h**) Schematic illustration of the internal organization of a single replication fork obtained from the TPC analysis. Straight and Dashed arrows represent regular and stalled replication processing, respectively.

Figure 4a shows a SR image of a U2OS cell nucleus with labeled nascent DNA (magenta), RPA (yellow) and PCNA (cyan). While repetitive features can be seen in individual foci (Fig. 4b), due to the abundance of the different factors, their specific spatial arrangement with respect to nascent DNA is barely discernable. By applying TPC analysis (Fig. 4c(i–iii), Supplementary Figure S6) we identified a clear correlation (triple-colocalization) of PCNA, RPA, and nascent DNA, which otherwise cannot be quantitatively distinguished from random colocalization incidents in such high-density images when interpreted via non-correlation methods. The TPC analyses enabled us to derive the average correlation distances of PCNA and RPA from nascent DNA as ~132 and ~150 nm, respectively. These metrics are significant as the observed patterns are anticipated based on current models describing replisome assembly^18^. We note that correlation distances are convolved with a number of factors that are intrinsic to single-molecule localization microscopy, such as the experimental localization accuracy (~15 nm, 37 nm, and 44 nm for Alexa Fluor 647, Alexa Fluor 568, and Alexa Fluor 488, respectively), volume of the antibodies used for immunofluorescence^19^,^20^, and the projection of 3D data to a 2D image. As such, the correlation distances cannot be used as absolute distance measurements, but rather provide an accurate description of the relative placement of molecules for analysis of their spatial arrangement. TPC analysis also revealed that the angle between DNA-PCNA and DNA-RPA axis is ~0 deg. (Fig. 4c(i,ii)). In addition to the fact that the observed cluster size is comparable with their correlation distance, we reasoned that the broad distribution of the correlation profile can arise from its 3D nature. To further test our analysis, we also imaged U2OS cells where nascent DNA, RPA, and the replicative helicase MCM were labeled. TPC analysis applied to these images yielded the same pattern, except that the correlation distance of MCM from nascent DNA was ~164 nm, which is further than PCNA and RPA (Supplementary Figure S8). This organization is in agreement with the prevailing replication fork model where the replicative DNA helicase is located at the front of the replication fork^18^.

Finally, we tested the ability of TPC to resolve perturbations in the arrangements of replisomes. To this end, we imaged S-phase U2OS cells that were treated with hydroxyurea (HU), which suppresses DNA replication by depleting dNTP pools^21^. Figure 4d shows the nucleus of a cell treated with 1 mM HU during replication, where a sharp decrease in the amount of nascent DNA (magenta) is observed. TPC analysis of HU-treated cells identified individual replication forks containing nascent DNA, PCNA, and RPA where PCNA and RPA are localized at the edge of nascent DNA clusters (Fig. 4f). The analysis revealed smaller nascent DNA clusters along with shorter DNA-PCNA and DNA-RPA distances of ~109 nm, and ~100 nm, respectively as compared to control cells (Fig. 4g,h, and Supplementary Figure S9).

In summary, we have demonstrated that TPC analysis is a robust approach, capable of unbiased identification of the spatial arrangement of distinct subpopulations of molecular complexes contained in noisy multicolor SR images. The fundamental strength of TPC analysis is its ability to distinguish every correlated three-component species from randomly distributed and uncorrelated populations, which would otherwise prevent quantitative analysis of the molecular complex of interest. As compared to Cross-Pair-Correlation analysis, TPC provides far more topological information by extending the measurable geometric properties from distance-only to distances together with their angular distributions and directionalities. We also note that the correlation algorithm presented in this study can be extended for unbiased analysis of 3D SR images. Consequently, the TPC approach is widely applicable for the comprehensive quantitative analysis of the spatial organization of molecular complexes in biological SR imaging, as well as other fields applying multiplexed high-resolution imaging.

## Methods

### Cell culture, cell synchronization, and *in vivo* EdU labeling

U2OS cells (ATCC HTB-96) were passaged onto glass coverslips and cultured in Modified McCoy’s 5A medium (ThermoFisher 16600) with 10% FBS (Germini Bio. 100–106) and 100 U/mL Penicillin-Streptomysin (ThermoFisher 15140) for 24 hours. The cells were then synchronized to early S-phase via 72 hours Serum withdrawal followed by 15 hours incubation in full medium^22^. The cells were incubated in full medium with 0 or 1 mM HU for another 2 hours, and during the last hour of HU treatment, EdU was added to a final concentration of 10 μM for nascent DNA labeling^23^.

### Imaging sample preparation

Cells were immediately permeablized and fixed after EdU labeling. In brief, cells were extracted with CSK buffer (10 mM Hepes, 300 mM Sucrose, 100 mM NaCl, 2 mM MgCl_2_, and 0.5% Triton X-100, pH = 7.4) for 10 minutes to remove replisome proteins that are unbound to chromatin, followed by fixation with paraformaldehyde (PFA, 4% in PBS) for 30 minutes^24^,^25^. Cells were then washed twice with blocking buffer (2% glycine, 2% BSA, 0.2% geltin, and 50 mM NH_4_Cl in PBS) and blocked for 1 hour at room temperature or overnight at 4 °C for further immunofluorescence staining.

Nascent DNA staining was performed by tagging Alexa Fluor 647 picolyl azide onto EdU via the ‘click’ reaction^26^ (Click-iT chemistry, thermoFisher C10640). RPA, PCNA, and MCM were stained with validated monoclonal antibodies^27^,^28^,^29^. In detail, RPA was immunostained with a rabbit monoclonal against RPA70 (Abcam, ab79398) followed by secondary immunostaining with goat-anti-rabbit conjugated with Alexa Fluor 568 (ThermoFisher, A11036); PCNA and MCM were immunostained with Mouse monoclonal against PCNA (Abcam, Ab29) and MCM5 (Abcam, ab6154), respectively, followed by secondary immunostaining with goat-anti-mouse conjugated with alexa Fluor 488 (ThermoFisher, A11029).

Coverslips were mounted onto a microscope microfluidics chamber for SR imaging.

### Microscope and Super-resolution imaging

We used a custom-built optical imaging platform based on a Leica DMI 300 inverted microscope equipped with three laser lines: 473 nm laser line (Opto Engine LLC, MBL-473-300 mW), 532 nm laser line (OEM Laser Systems, MLL-III 200 mW), and 640 nm laser line (OEM Laser Systems, MLL-III 150 mW). All three laser lines were collimated and reflected into an HCX PL APO 100X NA = 1.47 OIL CORR TIRF objective by a multi-band dichroic (Chroma, zt405/488/532/640rpc-XT). Cell samples were excited in Highly inclined and Laminated Optical sheet (HILO) illumination mode^30^, and a 405 nm laser line (Applied Scientific Pro., SL-405 nm-300 mW) was employed to enhance photoswitching of fluorophores.

The emitted fluorescence was sequentially recorded. In brief, fluorescence of Alexa Fluor 568 and Alexa Fluor 647 were simultaneously collected through a dual-band bandpass filter (Chroma, CY3/CY5, 59007m) and subsequently split into two channels using a dichroic mirror (Semrock, FF660-Di02) in conjugation within a dual-view cube (Photometrics, DV2); fluorescence of Alexa Fluor 488 was afterwards recorded with the dual-band bandpass filter replaced by a narrow single-band filter (Semrock, FF01-531/40) via a filter wheel (ThorLabs, FW102C), and then was reflected onto one half side of the camera by the FF660 dichroic. All three colors were recorded at 512 × 512 pixels (~with configured pixel size of 160 × 160 nm^2^) using an Andor iXon+ 897 EMCCD camera at 33 Hz for 2000 frames^31^. The three channels were calibrated by matching the same fluorescent beads (ThermoFisher, T7279) observed in different channels via a polynominal morph-type mapping algorithm.

Super-resolution imaging buffer was composed of an oxygen scavenging system (1 mg/mL glucose oxidase, 0.02 mg/mL catalase, and 0.8% glucose in PBS) and 100 mM cycteamine (MEA)^4^. The oxygen scavenging solution was freshly mixed before injection into the sample chamber.

### Data analysis

Single-molecule localization was performed by QuickPalm with previously described parameter sets^32^. Specifically, single-molecule localization was determined with the QuickPalm parameter *minimum SNR* = 4, and the *maximum FWHM* = 4. The obtained (x, y) coordinates list of all the localization events was then written into a new image with pixel sizes of 10 × 10 nm^2^. The localization uncertainty 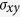 was evaluated through equation^33^ (3):


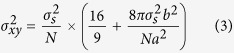


where 
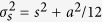
, *s* is the standard deviation of the PSF of each single molecule events, *a* is the pixel dimension; *N* is the number of photons and *b* is the background. The frequency counts of all uncertainties were then generated and the peak position given by a skew Gaussian fit of the frequency distribution was considered as the average uncertainty. For correlation analysis, the central region of each nucleus was cropped to avoid the heterogenic edge distribution effect^34^. The cropped region had a minimum dimension of 8 μm, substantially bigger than the scales of the complexes of interest (correlation distance ~100–200 nm).

Triple-Correlation was calculated using its Fourier Transforms known as bispectrum (equation (4)):


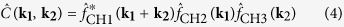


where 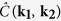 and 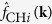 are the Fourier Transforms of *C*(**r**_1_, **r**_2_) and *ρ*_Ch*i*_(**r**), respectively, with **k**_*i*_ the corresponding spatial frequency of **r**_*i*_. Considering the computing difficulty for a direct 4D Inverse Fourier Transform with each dimension of ~800 pixels (or sampled frequency), we instead 2D scanned **r**_1_ (or **r**_2_), and accordingly performed 2D Fast Fourier Transform (FFT) and inverse FFT over **r**_2_ (or **r**_1_) at each fixed **r**_1_ (or **r**_2_) (Supplementary Note 2). The 4D Triple Correlation was then integrated through *θ*_1_ as mentioned in the main text and finally yielded the 3D correlation *g*(*r*_1_, *r*_2_, Δ*θ*). We integrated through each parameter of *g*(*r*_1_, *r*_2_, Δ*θ*) to yield a combination of three 2D correlation maps. For more precise quantification of each parameter, we integrated the 3D correlation data through the other two parameters and performed 1D fit via a modified Gaussian model (Supplementary Note 3).

## Additional Information

**How to cite this article**: Yin, Y. and Rothenberg, E. Probing the Spatial Organization of Molecular Complexes Using Triple-Pair-Correlation. *Sci. Rep*. **6**, 30819; doi: 10.1038/srep30819 (2016).

## Supplementary Material

Supplementary Information
