# Supplementary material for: Probing the Spatial Organization of Molecular Complexes Using Triple-Pair-Correlation
Source: Sci Rep. 2016 Aug 22;6:30819. doi: 10.1038/srep30819 (PMC4992864; doi:10.1038/srep30819)
Supplement: Supplementary Information [file srep30819-s1.pdf]

Supplementary Information for

Probing the Spatial Organization of Molecular Complexes Using Triple-Pair-Correlation

Yandong Yin, Eli Rothenberg*

Table of Contents

Supplementary Note 1 3
Supplementary Note 2 5
Supplementary Note 3 7
Supplementary Figure S1-S8 8

Supplementary Note 1

Consider in Channel 1 (CH1), a set of N_{CH1} point-like fluorophores at positions $\mathbf{R}_i^{\mathrm{CH1}}$ for $1 < i < N_{\mathrm{CH1}}$, in which each of them blinks stochastically M_{CH1} times on average and registers one *detection* for each blinking event. The probability density function of having one blinking event detected at location $\mathbf{R}$ is governed by the average localization uncertainty of the fluorophores in Channel 1 (σ_{CH1}) and written as:

$$p_{\mathrm{CH1}}(\mathbf{R}) = \frac{1}{N_{\mathrm{CH1}}} \sum_{i=1}^{N_{\mathrm{CH1}}} \frac{1}{\sqrt{2\pi}\sigma_{\mathrm{CH1}}} \exp\left[-\frac{\left(\mathbf{R}-\mathbf{R}_i^{\mathrm{CH1}}\right)^2}{2\sigma_{\mathrm{CH1}}^2}\right]$$

Accordingly, the average density of detections at location $\mathbf{R}$ in Channel 1 is given by:

$$\rho_{\mathrm{CH1}}(\mathbf{R}) = \frac{N_{\mathrm{CH1}}M_{\mathrm{CH1}}}{S} p_{\mathrm{CH1}}(\mathbf{R}) = \frac{M_{\mathrm{CH1}}}{S\sqrt{2\pi}\sigma_{\mathrm{CH1}}} \sum_{i=1}^{N_{\mathrm{CH1}}} \exp\left[-\frac{\left(\mathbf{R}-\mathbf{R}_i^{\mathrm{CH1}}\right)^2}{2\sigma_{\mathrm{CH1}}^2}\right]$$

where S is the area of interested, and $\langle\rho_{\mathrm{CH1}}\rangle_{\mathbf{R}} = N_{\mathrm{CH1}}M_{\mathrm{CH1}}/S$.

Therefore the average Triple-Pair-Correlation of Channel 1 (CH1), Channel 2 (CH2), and Channel 3 (CH3) is written as:

$$C(\mathbf{r}_1,\mathbf{r}_2) = \frac{1}{\langle\rho_{\mathrm{CH1}}\rangle_{\mathbf{R}}\langle\rho_{\mathrm{CH2}}\rangle_{\mathbf{R}}\langle\rho_{\mathrm{CH3}}\rangle_{\mathbf{R}}} \int \rho_{\mathrm{CH1}}(\mathbf{R})\rho_{\mathrm{CH2}}(\mathbf{R}+\mathbf{r}_1)\rho_{\mathrm{CH3}}(\mathbf{R}+\mathbf{r}_2)\mathrm{d}\mathbf{R}$$

$$= A \sum_{i=1}^{N_{\mathrm{CH1}}} \sum_{j=1}^{N_{\mathrm{CH2}}} \sum_{k=1}^{N_{\mathrm{CH3}}} \int \mathrm{d}\mathbf{R} \exp\left[-\frac{\left(\mathbf{R}-\mathbf{R}_i^{\mathrm{CH1}}\right)^2}{2\sigma_{\mathrm{CH1}}^2}\right] \exp\left[-\frac{\left(\mathbf{R}+\mathbf{r}_1-\mathbf{R}_j^{\mathrm{CH2}}\right)^2}{2\sigma_{\mathrm{CH2}}^2}\right] \exp\left[-\frac{\left(\mathbf{R}+\mathbf{r}_2-\mathbf{R}_k^{\mathrm{CH3}}\right)^2}{2\sigma_{\mathrm{CH3}}^2}\right]$$

$$= A \sum_{i=1}^{N_{\mathrm{CH1}}} \sum_{j=1}^{N_{\mathrm{CH2}}} \sum_{k=1}^{N_{\mathrm{CH3}}} \int \mathrm{d}\mathbf{R} \exp\left[-\frac{\mathbf{R}^2}{2\sigma_{\mathrm{CH1}}^2}\right] \exp\left[-\frac{\left(\mathbf{R}+\mathbf{r}_1-\Delta\mathbf{r}_{ij}\right)^2}{2\sigma_{\mathrm{CH2}}^2}\right] \exp\left[-\frac{(\mathbf{R}+\mathbf{r}_2-\Delta\mathbf{r}_{ik})^2}{2\sigma_{\mathrm{CH3}}^2}\right]$$

where $A = 1/\left(2\pi^{3/2}\sigma_{\mathrm{CH1}}\sigma_{\mathrm{CH2}}\sigma_{\mathrm{CH3}}N_{\mathrm{CH1}}N_{\mathrm{CH2}}N_{\mathrm{CH3}}\right)$ and $\Delta\mathbf{r}_{ij} = \mathbf{R}_j^{\mathrm{CH2}} - \mathbf{R}_i^{\mathrm{CH1}}, \Delta\mathbf{r}_{ik} = \mathbf{R}_k^{\mathrm{CH3}} - \mathbf{R}_i^{\mathrm{CH1}}$.

For simplicity, define $f_{\mathrm{CH1}}(\mathbf{R}) = \exp[-\mathbf{R}^2/(2\sigma_{\mathrm{CH1}}^2)]$, and $C(\mathbf{r}_1,\mathbf{r}_2)$ is then written as:

$$C(\mathbf{r}_1,\mathbf{r}_2) = A \sum_{i=1}^{N_{\mathrm{CH1}}} \sum_{j=1}^{N_{\mathrm{CH2}}} \sum_{k=1}^{N_{\mathrm{CH3}}} \int \mathrm{d}\mathbf{R} f_{\mathrm{CH1}}(\mathbf{R}) f_{\mathrm{CH2}}\left(\mathbf{R}+\mathbf{r}_1-\Delta\mathbf{r}_{ij}\right) f_{\mathrm{CH3}}(\mathbf{R}+\mathbf{r}_2-\Delta\mathbf{r}_{ik})$$

and its Fourier Transfer (Supplementary Note 2) is

$$\hat{C}(\mathbf{k}_1,\mathbf{k}_2) = A \sum_{i=1}^{N_{\mathrm{CH1}}} \sum_{j=1}^{N_{\mathrm{CH2}}} \sum_{k=1}^{N_{\mathrm{CH3}}} \hat{f}_{\mathrm{CH1}}^{conj}(\mathbf{k}_1+\mathbf{k}_2) \hat{f}_{\mathrm{CH2}}(\mathbf{k}_1) \hat{f}_{\mathrm{CH3}}(\mathbf{k}_2) \exp\left[-2\pi i\left(\Delta\mathbf{r}_{ij}\cdot\mathbf{k}_1 + \Delta\mathbf{r}_{ik}\cdot\mathbf{k}_2\right)\right]$$

$$= A \cdot \hat{C}_\sigma(\mathbf{k}_1,\mathbf{k}_2) \sum_{i=1}^{N_{\mathrm{CH1}}} \sum_{j=1}^{N_{\mathrm{CH2}}} \sum_{k=1}^{N_{\mathrm{CH3}}} \hat{C}_{ijk}(\mathbf{k}_1,\mathbf{k}_2)$$

where $\hat{C}_\sigma(\mathbf{k}_1,\mathbf{k}_2)$ is the Fourier Transform of the triple-convolution $C_\sigma(\mathbf{r}_1,\mathbf{r}_2)$ of the localization uncertainties from the three channels, and defined as $C_\sigma(\mathbf{r}_1,\mathbf{r}_2) = \int \mathrm{d}\mathbf{R} f_{\mathrm{CH1}}(\mathbf{R}) f_{\mathrm{CH2}}(\mathbf{R}+\mathbf{r}_1) f_{\mathrm{CH3}}(\mathbf{R}+\mathbf{r}_2)$, representing how localization uncertainties

contribute to the final triple-pair-correlation; and $\hat{C}_{ijk}(\mathbf{k}_1,\mathbf{k}_2)$ is the Fourier Transform of the spatial triple-correlation $C_{ijk}(\mathbf{r}_1,\mathbf{r}_2)$ of the fluorophores in the three channels, with its Fourier Transform $\hat{C}_{ijk}(\mathbf{k}_1,\mathbf{k}_2) = \exp\left[-2\pi i\left(\Delta\mathbf{r}_{ij} \cdot \mathbf{k}_1 + \Delta\mathbf{r}_{ik} \cdot \mathbf{k}_2\right)\right]$, representing how spatially correlated of the fluorophores in the three different channels.

Finally, $C(\mathbf{r}_1,\mathbf{r}_2)$ can be written as the inverse Fourier Transform of $\hat{C}(\mathbf{k}_1,\mathbf{k}_2)$:

$$C(\mathbf{r}_1,\mathbf{r}_2) = \mathcal{F}^{-1}\left(\hat{C}(\mathbf{k}_1,\mathbf{k}_2)\right) = A \cdot C_\sigma(\mathbf{r}_1,\mathbf{r}_2) * \sum_{ijk} C_{ijk}(\mathbf{r}_1,\mathbf{r}_2)$$

where $\mathcal{F}^{-1}$denotes the inverse Fourier Transfer and $*$ denotes a convolution operator.

Supplementary Note 2

Triple-Correlation function calculates the average probability for simultaneously observing three molecules, each in a different channel, as a function of their relative displacement, given by:

$$C(\mathbf{r_1},\mathbf{r_2}) = \frac{1}{\langle\rho_{\text{CH1}}\rangle_{\mathbf{R}}\langle\rho_{\text{CH2}}\rangle_{\mathbf{R}}\langle\rho_{\text{CH3}}\rangle_{\mathbf{R}}}\int \rho_{\text{CH1}}(\mathbf{R})\rho_{\text{CH2}}(\mathbf{R}+\mathbf{r_1})\rho_{\text{CH3}}(\mathbf{R}+\mathbf{r_2})\text{d}\mathbf{R} \quad (1)$$

where $\rho_{\text{CH}i}(\mathbf{R})$ is the surface density of detections at position $\mathbf{R} = |R,\theta\rangle$ in channel i. $\langle\ \rangle_{\mathbf{R}}$ denotes averaging operation over $\mathbf{R}$. The Fourier Transform of the Triple-Correlation, known as its bispectrum is given by:

$$\hat{C}(\mathbf{k_1},\mathbf{k_2}) = \hat{\rho}_{\text{CH1}}^{conj}(\mathbf{k_1}+\mathbf{k_2})\hat{\rho}_{\text{CH2}}(\mathbf{k_1})\hat{\rho}_{\text{CH3}}(\mathbf{k_2})$$

where $\hat{C}(\mathbf{k_1},\mathbf{k_2})$ and $\hat{\rho}_{\text{CH}i}(\mathbf{k})$ are the Fourier Transforms of $C(\mathbf{r_1},\mathbf{r_2})$ and $\rho_{\text{CH}i}(\mathbf{r})$, respectively, with $\mathbf{k}_i$ the corresponding spatial frequency of $\mathbf{r}_i$. $C(\mathbf{r_1},\mathbf{r_2})$ is calculated via an Inverse Fourier Transform from $\hat{C}(\mathbf{k_1},\mathbf{k_2})$, which involves a 4D FFT operation with each dimension of ~ 800 elements, and goes far beyond the computing capability of most current cluster computers. To address the *insufficient RAM* problem and conduct the computation on a desktop computer we implemented the following indirect procedure:

According to the definition of the Triple-Correlation per Equation (1), at each fixed $\mathbf{r_1}$, this can be written as:

$$C(\mathbf{r_2}|\mathbf{r_1}) = \frac{1}{\langle\rho_{\text{CH1}}\rangle_{\mathbf{R}}\langle\rho_{\text{CH2}}\rangle_{\mathbf{R}}\langle\rho_{\text{CH3}}\rangle_{\mathbf{R}}}\int \rho_{\text{CH12}}(\mathbf{R}|\mathbf{r_1})\rho_{\text{CH3}}(\mathbf{R}+\mathbf{r_2})\text{d}\mathbf{R} \quad (2)$$

with $\rho_{\text{CH12}}(\mathbf{R}|\mathbf{r_1}) = \rho_{\text{CH1}}(\mathbf{R})\rho_{\text{CH2}}(\mathbf{R}+\mathbf{r_1})$ being a function of a single variable $\mathbf{R}$. The Fourier Transform of Equation (2) can therefore be derived as:

$$\hat{C}(\mathbf{k_2}|\mathbf{r_1}) = \hat{\rho}_{\text{CH3}}(\mathbf{k_2})\hat{\rho}_{\text{CH1}}^{conj}(\mathbf{k_2}|\mathbf{r_1}) \quad (3)$$

and a 2D Triple-Correlation map at a fixed $\mathbf{r_1}$ (Equation (2)) can be subsequently calculated by inverse Fourier Transform of Equation (3). We then carried a 2D scan of $\mathbf{r_1}$, yielding the 4D map of the Triple-Correlation $C(r_1,\theta_1,r_2,\theta_2=\theta_1+\Delta\theta)$ (Main Text), which was then integrated along θ_1 to generate the final 3D $g(r_1,r_2,\Delta\theta)$ cube.

We note that during scanning of $\mathbf{r_1}$, $\rho_{\text{CH2}}(\mathbf{R}+\mathbf{r_1})$ can co-localize with $\rho_{\text{CH3}}(\mathbf{R})$ at a specific $\mathbf{r_1}$, and consequentially result in a co-localization between the product $\rho_{\text{CH12}}(\mathbf{R}|\mathbf{r_1})$ and $\rho_{\text{CH3}}(\mathbf{R})$. We also note that, at this $\mathbf{r_1}$, value, $\rho_{\text{CH12}}(\mathbf{R}|\mathbf{r_1}) = \rho_{\text{CH1}}(\mathbf{R})\rho_{\text{CH2}}(\mathbf{R}+\mathbf{r_1})$ stays non-zero, especially in a highly dense image, due to the random co-localization between $\rho_{\text{CH1}}(\mathbf{R})$ and $\rho_{\text{CH2}}(\mathbf{R}+\mathbf{r_1})$. Therefore, the specific co-localization between the non-zero $\rho_{\text{CH12}}(\mathbf{R}|\mathbf{r_1})$ and $\rho_{\text{CH3}}(\mathbf{R})$ can result in a residual correlation signal at $\mathbf{r_2}=0$ at this specific $\mathbf{r_1}$, such as the correlation at $\mathbf{r}_{\text{M-Y}}=10$, and $\mathbf{r}_{\text{M-C}}=0$ in Figure 2d(iii) in the main text. However, since in this case the $\rho_{\text{CH12}}(\mathbf{R}|\mathbf{r_1})$ represents random co-localizations between $\rho_{\text{CH1}}(\mathbf{R})$ and $\rho_{\text{CH2}}(\mathbf{R}+\mathbf{r_1})$, this residual signal is insignificant compared to the true correlation (e.g. the correlation at $\mathbf{r}_{\text{M-Y}}=10$, $\mathbf{r}_{\text{M-C}}=20$ in Figure 2d(iii)), and can be easily excluded by the $\mathbf{r_2}-\Delta\theta$ correlation map (e.g. Figure 2d(ii)).

To improve the accessibility of the Triple Correlation cube, we further integrate $g(r_1, r_2, \Delta\theta)$ along each dimension to make it a combination of three 2D correlation maps (Fig. 1c). Specifically, we transformed r_1-$\Delta\theta$ and r_2-$\Delta\theta$ maps from Cartesian coordinates to Polar coordinates via 2D cubic interpolation algorithm (MATLAB, MathWorks).

All three maps were then further integrated along each dimension so that r_1, r_2, and $\Delta\theta$ can be fitted into a 1D modified Gaussian profile (Supplementary Note 3).

Supplementary Note 3

The 1D correlation profile as a function of r_1 (or r_2) was obtained via integrating $g(r_1, r_2, \Delta\theta)$ along r_2 (or r_1) and $\Delta\theta$, with the latter performed radially through $[-\pi, \pi]$

$$g_{r_1}(r_1) = \int_{-\pi}^{\pi} \mathrm{d}\Delta\theta \int g(r_1, r_2, \Delta\theta)\mathrm{d}r_2$$

$$g_{r_2}(r_2) = \int_{-\pi}^{\pi} \mathrm{d}\Delta\theta \int g(r_1, r_2, \Delta\theta)\mathrm{d}r_1$$

The 1D correlation profile $g_{r_1}(r_1)$ (or $g_{r_2}(r_2)$) was approximated via a modified1D Gaussian distribution as a function of r_1 (or r_2)

$$g_r(r > 0) = \frac{A}{\sqrt{2\pi}\sigma} exp\left(-\frac{(r - r_0)^2}{2\sigma^2}\right) + \frac{A}{\sqrt{2\pi}\sigma} exp\left(-\frac{(r + r_0)^2}{2\sigma^2}\right) \qquad (4)$$

where $r_0 > 0$ and $\sigma > 0$ denotes the center and the standard deviation of the Gaussian function, respectively. Note that in Polar coordinates, a Gaussian distribution cannot extend to the $r < 0$ region. Instead, the '$r < 0$' portion of a normal Gaussian distribution centering at $r = r_0 > 0$ is considered as the '$r > 0$' portion of another normal Gaussian distribution centering at $r = -r_0 < 0$. Therefore, the radial integration along $\Delta\theta$ through $[-\pi, \pi]$ yields a modified 1D Gaussian profile (Equation (4)) where the first and the second terms originate from the $r > 0$ portion of a normal Gaussian distribution centered at $r = r_0 > 0$ and another Gaussian distribution centered at $r = -r_0 < 0$, respectively.

The correlation distance was then calculated as follows:

$$d = \frac{\int_0^\infty g_r(r) r \mathrm{d}r}{\int_0^\infty g_r(r)\mathrm{d}r} \qquad (5)$$

The 1D correlation profile as a function of $\Delta\theta$ was obtained via integrating $g(r_1, r_2, \Delta\theta)$ along r_2 and r_1

$$g_{\Delta\theta}(\Delta\theta) = \iint g(r_1, r_2, \Delta\theta)\mathrm{d}r_1\mathrm{d}r_2$$

Since the molecular pattern can be imaged as a pair of reflectional symmetries, with $g_{\Delta\theta}(\Delta\theta)$ being symmetric with respect to $\Delta\theta = 0$, we used a modified Gaussian distribution similar to Equation (4)

$$g_{\Delta\theta}(\Delta\theta) = \frac{A}{\sqrt{2\pi}\sigma} exp\left(-\frac{(\Delta\theta - \Delta\theta_0)^2}{2\sigma^2}\right) + \frac{A}{\sqrt{2\pi}\sigma} exp\left(-\frac{(\Delta\theta + \Delta\theta_0)^2}{2\sigma^2}\right) \qquad (6)$$

where $\Delta\theta_0 > 0$ and $\sigma > 0$ denotes the center and the standard deviation of the Gaussian function, respectively.

Supplementary Figure S1-S8

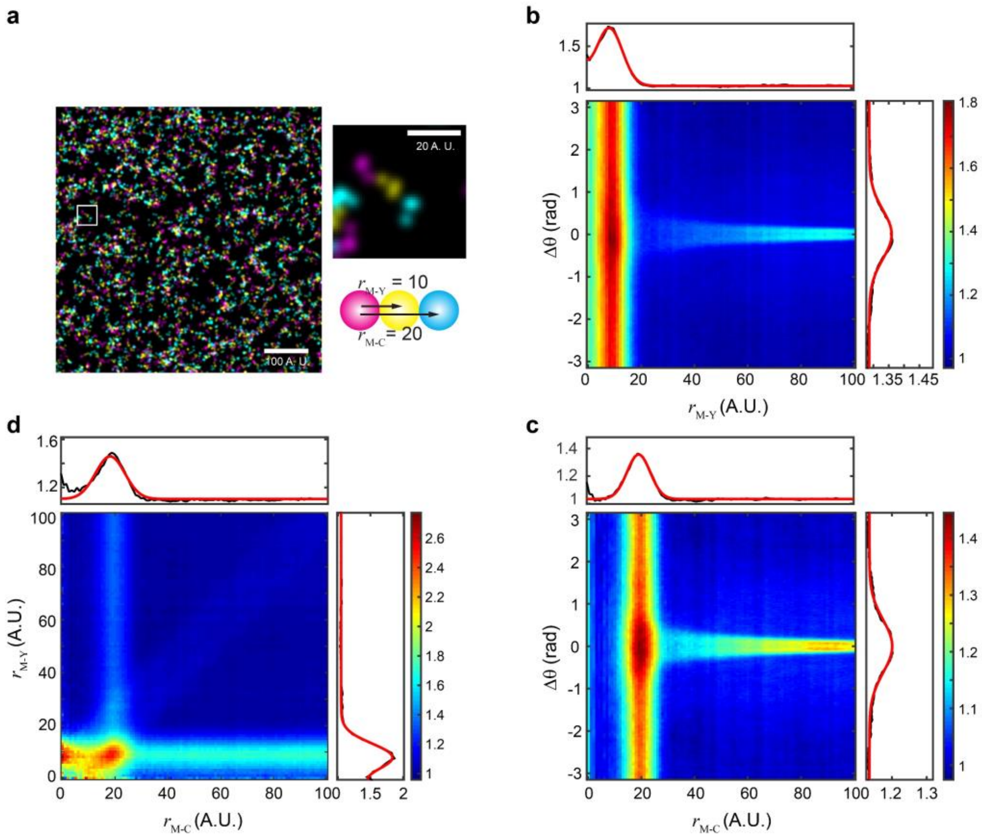

Supplementary Figure S1. Correlation profile is well fitted with modified Gaussian function. (**a**) Image of simulated molecular pattern (left), with a zoomed-in image shown on the right along with the geometric features of the internal arrangement of the molecular pattern. (**b**) A 2D Triple-Correlation map as a function of r_{M-Y} and $\Delta\theta$, and its 1D correlation $g_{r_{M-Y}}(r)$ obtained by integrating the 2D map along $\Delta\theta$ (i, black line); its 1D correlation $g_{\Delta\theta}(\Delta\theta)$ obtained by integrating the 2D map along r_{M-Y} (ii, black line). Both of these are well fitted by Equation (4) and (6), (red lines, Supplementary Note 3) yielding a correlation distance of $r_{M-Y} = 8.33 \pm 0.07$ A.U. (Equation (5) in Supplementary Note 3) and Gaussian distribution of $\Delta\theta$ centered at 0.00 with $\sigma = 32.1$ deg. (**c**) A 2D Triple-Correlation map as a function of r_{M-C} and $\Delta\theta$, and its 1D correlation $g_{r_{M-C}}(r)$ (i, black line) and $g_{\Delta\theta}(\Delta\theta)$ (ii, black lines). Their fits (red lines) yield a correlation distance of $r_{M-C} = 19.4 \pm 0.2$ A.U and a Gaussian distribution of $\Delta\theta$ centering at 0.00 with $\sigma = 34.9$ deg. (**d**) A 2D Triple-Correlation map as a function of r_{M-Y} and r_{M-Y}, and its 1D correlation $g_{r_{M-Y}}(r)$ (i, black line) and $g_{r_{M-C}}(r)$ (ii, black lines). Their fits (red lines) yield the correlation distance of $r_{M-Y} = 8.31 \pm 0.06$ and $r_{M-C} = 18.4 \pm 0.5$. These results are all in excellent agreement with the simulated molecular patterns in terms of their internal spatial organization. The Triple-Correlation is an average of Triple-Correlation profiles 10 simulated images. Errors are given as propagated fitting errors.

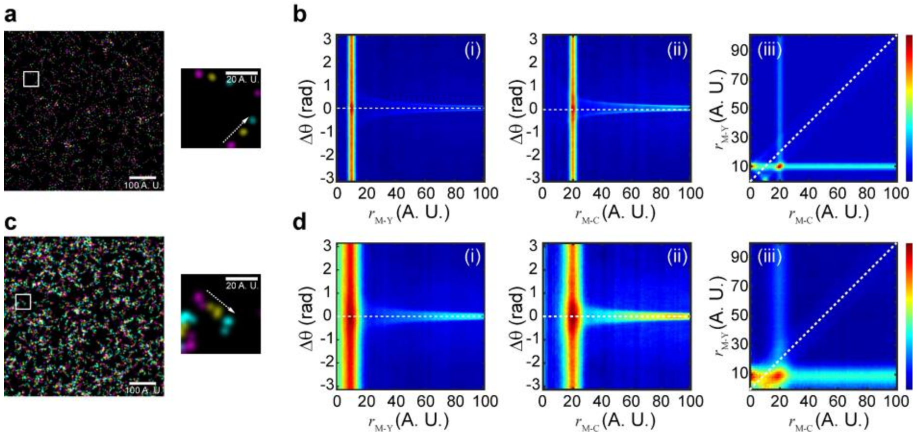

Supplementary Figure S2. Localization uncertainty widens TPC profiles. (**a** and **c**) Simulated molecular patterns with the same internal geometry properties but different localization uncertainties. The simulated localization uncertainty is $\sigma_{CH1} = \sigma_{CH2} = \sigma_{CH3} = 1\ \mathrm{AU}$ and $\sigma_{CH1} = \sigma_{CH2} = \sigma_{CH3} = 3\ \mathrm{AU}$ for (a) and (c), respectively. (**b** and **d**) Normalized 2D Triple-Correlation maps derived from simulated patterns in (a) and (c). A higher level of localization uncertainty (c) results in widened TPC profiles.

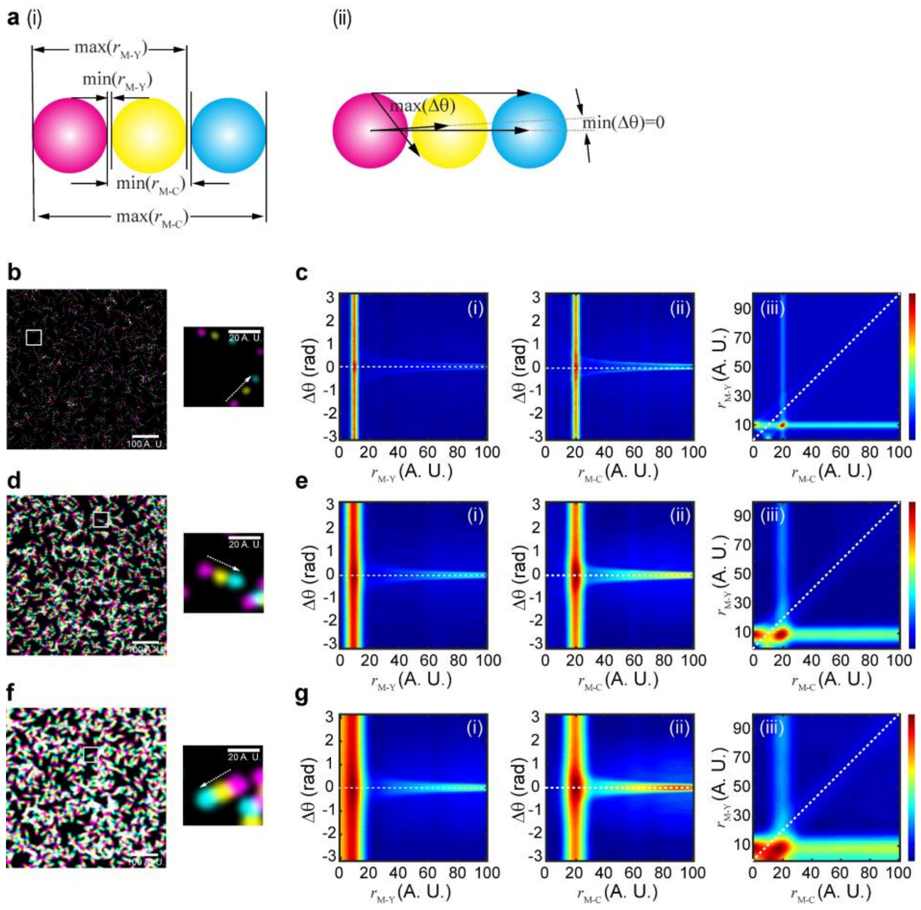

Supplementary Figure S3. A broad distribution along $\mathbf{r}_{\mathbf{M-Y}}$, $\mathbf{r}_{\mathbf{M-C}}$, and $\Delta\boldsymbol{\theta}$ results from cluster sizes comparable to their inter-cluster distances. (**a**) Schematic illustration of how cluster size broadens the TPC profile along $\mathbf{r}_{\mathrm{M-Y}}$ **and** $\mathbf{r}_{\mathrm{M-C}}$ (i), and $\Delta\theta$ (ii). (**b**, **d**, and **f**) Simulated molecular patterns of the same linear and sequential internal organization but with different cluster size of $R = 1$, 3, and 5 units for (b), (d), and (f), respectively. (**c**, **e**, and **g**) Normalized 2D Triple-Correlation maps derived from simulated patterns in (b, d, and f). The molecular patterns with a smaller cluster size results in a narrower correlation profile along r_{M-Y}, r_{M-C}, and $\Delta\theta$.

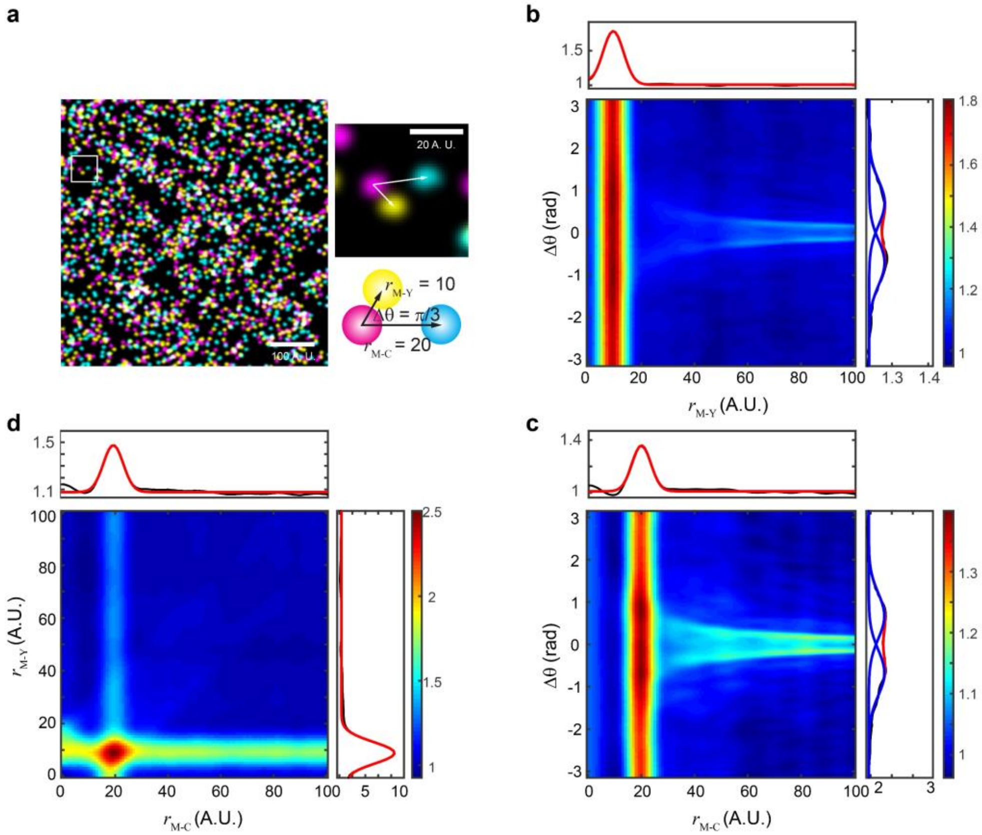

Supplementary Figure S4. Triple-Correlation profile of triangular molecular patterns is well fitted with a modified Gaussian function. (**a**) Image of a simulated triangular molecular pattern (left), with a zoomed-in image shown on the right along with the geometric features of a molecular pattern internal arrangement. (**b**) A 2D Triple-Correlation map as a function of r_{M-Y} and $\Delta\theta$, and its 1D correlation $g_{r_{M-Y}}(r)$ (i, black line) and $g_{\Delta\theta}(\Delta\theta)$ (ii, black line). Their fits (Equation (4) and (6), red lines, Supplementary Note 3) yield a correlation distance of $r_{M-Y} = 9.08 \pm 0.04$ A.U. (Equation (5) in Supplementary Note 3) and a Gaussian distribution of $\Delta\theta$ centered at 42.0 with $\sigma = 35.0$ deg. (**c**) A 2D Triple-Correlation map as a function of r_{M-C} and $\Delta\theta$, and its 1D correlation $g_{r_{M-C}}(r)$ (i, black line) and $g_{\Delta\theta}(\Delta\theta)$ (ii, black lines). Their fits (red lines) yield a correlation distance of $r_{M-C} = 19.7 \pm 0.1$ A.U and a Gaussian distribution of $\Delta\theta$ centered at 38.4 with $\sigma = 32.7$ deg. (**d**) A 2D Triple-Correlation map as a function of r_{M-Y} and r_{M-Y}, and its 1D correlation $g_{r_{M-Y}}(r)$ (i, black line) and $g_{r_{M-C}}(r)$ (ii, black lines). Their fits (red lines) yield correlation distances of $r_{M-Y} = 9.09 \pm 0.04$ and $r_{M-C} = 19.5 \pm 0.2$. The resolved distances are in excellent agreement with the simulated molecular patterns in terms of their internal spatial organization, but the resolved $\Delta\theta$ display a relative discrepancy with respect to their pre-assigned parameters. We reason that this discrepancy stemmed from the 2D cubic interpolation from Cartesian to Polarized coordinates as mentioned in Supplementary Note 2. The Triple-Correlation is an average of Triple-Correlation profiles from 10 simulated images. Errors are given as propagated fitting errors.

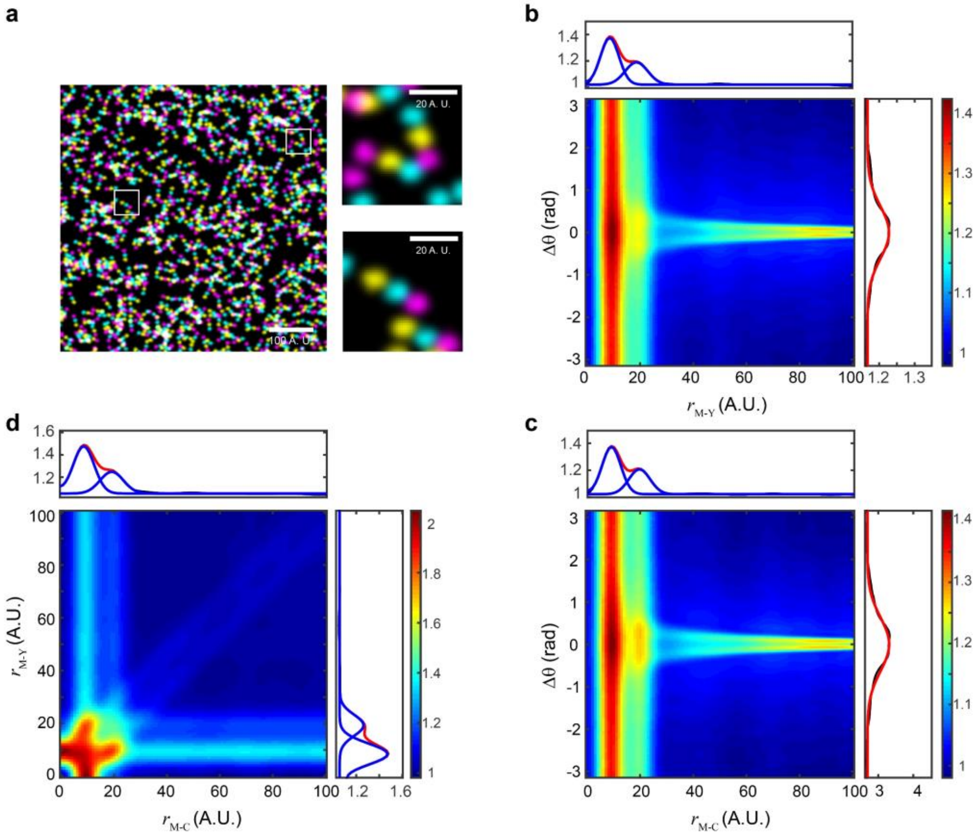

Supplementary Figure S5. Different molecular patterns are revealed by Triple-Correlation. (**a**) Image of simulated molecular patterns (left), with a zoomed-in image shown on the right along with the geometric features of the molecular patterns' internal arrangement. The two different patterns are randomly distributed in the image. (**b**) A 2D Triple-Correlation map as a function of r_{M-Y} and $\Delta\theta$, and its 1D correlation $g_{r_{M-Y}}(r)$ (i, black lines) and $g_{\Delta\theta}(\Delta\theta)$ (ii, black line). Both of these are well fitted (Equation (4) and Equation (6), red lines, Supplementary Note 3). $g_{r_{M-Y}}(r)$ is fitted with two Gaussian distributions which yield a correlation distances of $r_{M-Y\_1} = 9.00 \pm 0.12$ A.U. and $r_{M-Y\_2} = 19.1 \pm 0.3$ (Equation (5) in Supplementary Note 3); $g_{\Delta\theta}(\Delta\theta)$ yields a Gaussian distribution of $\Delta\theta$ centered at 0.00 with $\sigma = 35.0$ deg. (**c**) A 2D Triple-Correlation map as a function of r_{M-C} and $\Delta\theta$, and its 1D correlation $g_{r_{M-C}}(r)$ (i, black line) and $g_{\Delta\theta}(\Delta\theta)$ (ii, black lines). Similarly, $g_{r_{M-Y}}(r)$ is fitted with two Gaussian distributions, yielding correlation distances of $r_{M-C\_1} = 8.92 \pm 0.10$ A.U. and $r_{M-C\_2} = 19.4 \pm 0.2$; $g_{\Delta\theta}(\Delta\theta)$ yields a Gaussian distribution of $\Delta\theta$ centered at 0.00 with $\sigma = 35.5$ deg. (**d**) A 2D Triple-Correlation map as a function of r_{M-Y} and r_{M-Y}, and its 1D correlation $g_{r_{M-Y}}(r)$ (i, black line) and $g_{r_{M-C}}(r)$ (ii, black lines). These fits yield correlation distances of $r_{M-Y\_1} = 8.8 \pm 0.2$, $r_{M-C\_1} = 19.4 \pm 0.4$ and $r_{M-Y\_2} = 8.8 \pm 0.1$, $r_{M-C\_2} = 19.6 \pm 0.2$. The two different patterns appear as two distinct distributions. The Triple-Correlation is an average of Triple-Correlation profiles from 10 simulated images. Errors are given as propagated fitting errors.

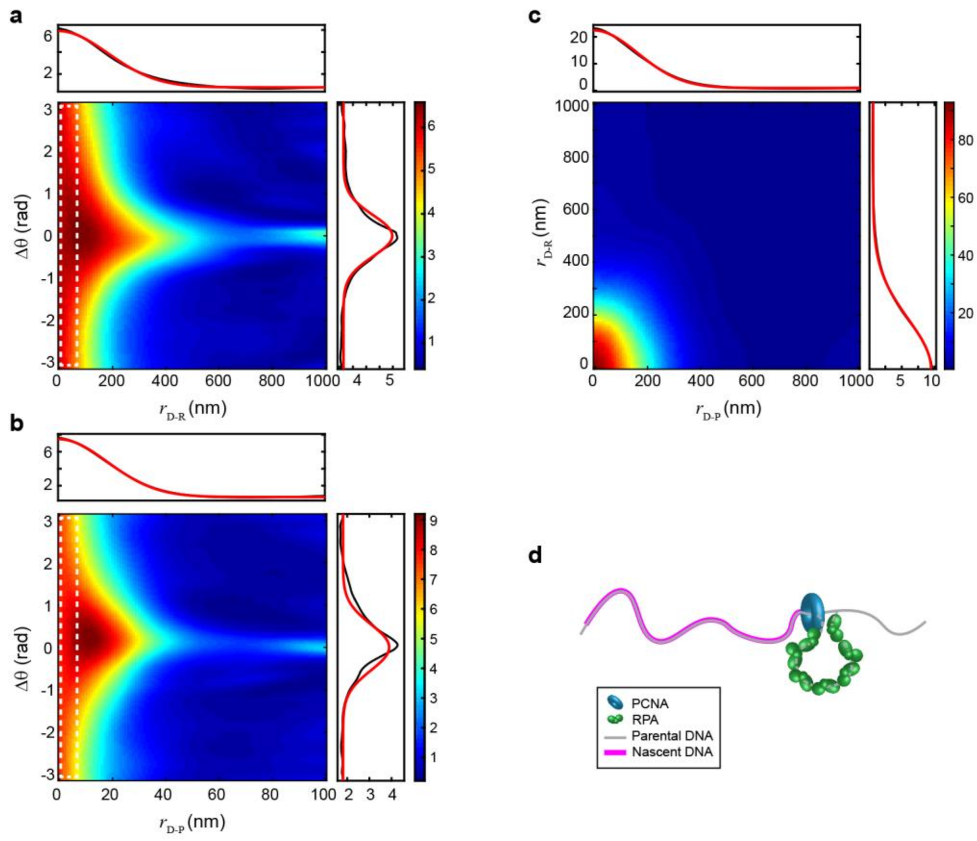

Supplementary Figure S6. Spatial organization of nascent DNA, PCNA, and RPA within replication fork foci is revealed by Triple-Correlation. (**a**) A 2D Triple-Correlation map as a function of the distance between DNA and RPA (r_{D-R}) and $\Delta\theta$, and its 1D correlation $g_{r_{D-R}}(r)$ (i, black line) and $g_{\Delta\theta}(\Delta\theta)$ (ii, black line). Both curves are well fitted with Equation (4) and (6) (red lines, Supplementary Note 3) yielding $r_0 = 13 \pm 17$ nm with $\sigma = 186 \pm 1$ nm, and consequently a correlation distance of $r_{D-R} = 148.3 \pm 1.3$ nm (Equation (5) in Supplementary Note 3);and a Gaussian distribution of $\Delta\theta$ centered at 0.00 with $\sigma = 28.1$ deg. (**b**) A 2D Triple-Correlation map as a function of distance between DNA and PCNA (r_{D-P}) and $\Delta\theta$, and its 1D correlation $g_{r_{D-P}}(r)$ (i, black line) and $g_{\Delta\theta}(\Delta\theta)$ (ii, black lines). Their fits (red lines) yielded $r_0 = 24 \pm 4$ nm with $\sigma = 184.9 \pm 0.6$ nm, and consequently a correlation distance of $r_{D-P} = 147.2 \pm 0.5$ nm and a Gaussian distribution of $\Delta\theta$ centered at 0.00 with $\sigma = 24.6$ deg. (**c**) A 2D Triple-Correlation map as a function of r_{D-R} and r_{D-P}, and its 1D correlation $g_{r_{D-R}}(r)$ (i, black line) and $g_{r_{D-P}}(r)$ (ii, black lines). These fits (red lines) yield correlation distances of $r_{D-R} = 150.9 \pm 0.5$ nm (with $r_0 = 25 \pm 4$, $\sigma = 187.8 \pm 0.6$, Equation 4), and $r_{D-P} = 133.4 \pm 0.6$ nm (with $r_0 = 25 \pm 5$, $\sigma = 166 \pm 1$, Equation 4). The Triple-Correlation is an average of Triple-Correlation profiles of 13 nuclei in U2OS cells. Errors are given as propagated fitting errors. We note that the overlapped portion between nascent DNA and replisome proteins resulted in a high Triple-Correlation response at r_{D-R} (or r_{D-P}) of ~ 0 nm through all $\Delta\theta = [-\pi, \pi]$, and further resulted in high correlation at r_{D-R} (or r_{D-P}) of ~ 0 nm, as represented in the 1D correlation plots after integration of $\Delta\theta$ (Supplementary Figure S7 and Supplementary Note 3) For the order of integration, we found that when the 2D interpolation from Cartesian to Polar coordinates (Cubic algorithm, Supplementary Note 1) is followed by integration of $\Delta\theta$ does not converge to accurately represent the correlation distance (c). Therefore, we take the correlation distance obtained in (c), which integrates $\Delta\theta$ without 2D interpolation beforehand, as more accurate correlation distance. (**d**)

Schematic illustration of the spatial organization of nascent DNA, RPA, and PCNA within a replication fork derived from the Triple Correlation analysis.

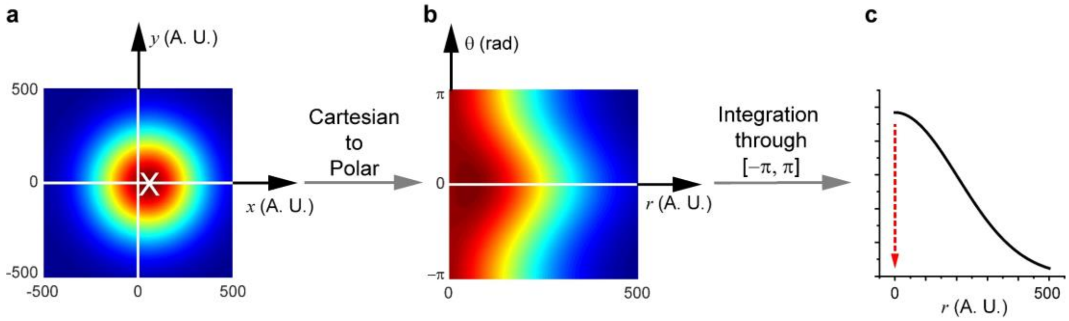

Supplementary Figure S7. Broad 2D correlation profile in Cartesian coordinates results in a high correlation distribution at $r = 0$ nm. (**a**) A schematic illustration of a 2D correlation profile in Cartesian Coordinates, with its center close, but not equals to zero, and a quite broad distribution. (**b**) the 2D correlation profile obtained by transforming the correlation in (a) from Cartesian to Polar coordinates. Due to its non-zero center and broad distribution, transforming from a Cartesian to Polar coordinate system yields high correlation at $r = 0$ nm contributing to all $[-\pi, \pi]$ distributions. (**c**) 1D correlation plots obtained by integrating the correlation in (b) (or (a)) through $[-\pi, \pi]$. Due to the high correlation at $r = 0$ nm all through $[-\pi, \pi]$, the 1D correlation plot peaks at $r = 0$ rather than at its original center represented in (a).

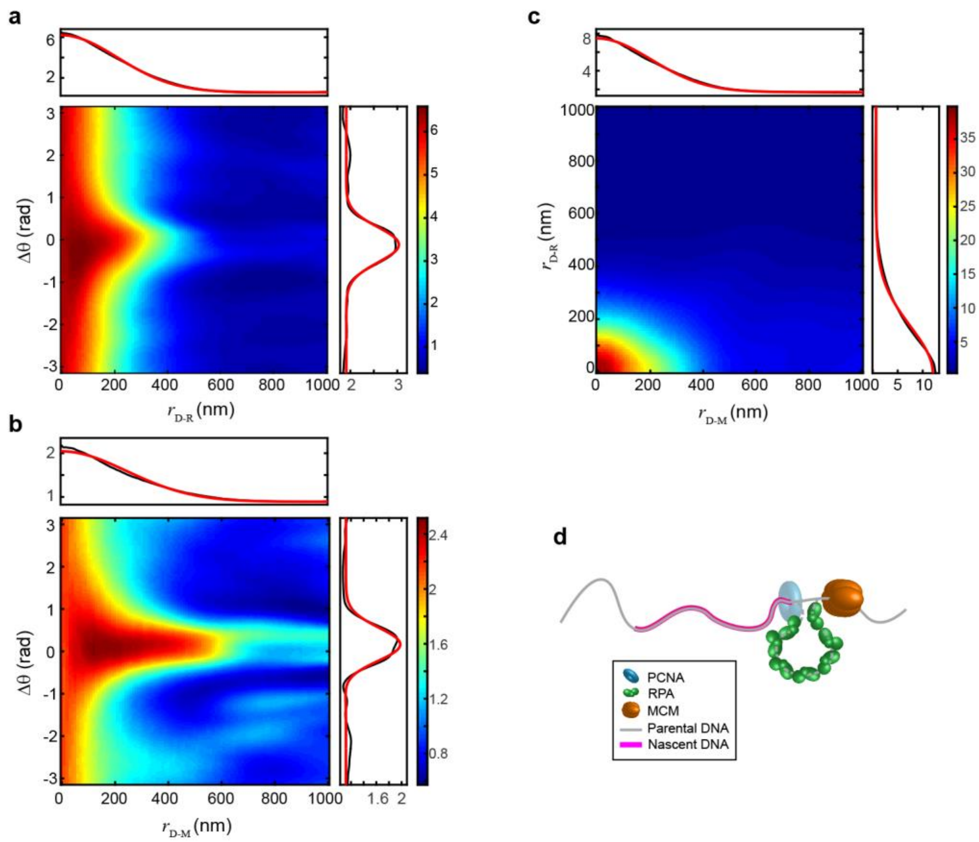

Supplementary Figure S8. Spatial organization of nascent DNA, MCM, and RPA within replication fork foci is revealed by Triple-Correlation. (**a**) A 2D Triple-Correlation map as a function of distance between DNA and RPA (r_{D-R}) and $\Delta\theta$, and its 1D correlation $g_{r_{D-R}}(r)$ (i, black line) and $g_{\Delta\theta}(\Delta\theta)$ (ii, black line), with the latter yielding a Gaussian distribution of $\Delta\theta$ centered at 0.00 with $\sigma = 24.6$ deg. (**b**) A 2D Triple-Correlation map as a function of the distance between DNA and MCM (r_{D-M}) and $\Delta\theta$, and its 1D correlation $g_{r_{D-M}}(r)$ (i, black line) and $g_{\Delta\theta}(\Delta\theta)$ (ii, black lines), with the latter yielding a Gaussian distribution of $\Delta\theta$ centered at 0.00 with $\sigma = 24.6$ deg. (**c**) A 2D Triple-Correlation map as a function of r_{D-R} and r_{D-M}, and its 1D correlation $g_{r_{D-R}}(r)$ (i, black line) and $g_{r_{D-M}}(r)$ (ii, black lines). Their fits (red lines) yield correlation distances of $r_{D-R} = 141.4 \pm 1.0$ nm (with $r_0 = 25 \pm 9$, $\sigma = 175.9 \pm 1.3$, Equation 4), and $r_{D-M} = 165.1 \pm 1.0$ nm (with $r_0 = 25 \pm 11$, $\sigma = 205.7 \pm 1.3$, Equation 4). The Triple-Correlation is an average of the Triple-Correlation profiles of 9 nuclei in U2OS cells. Errors are given as propagated fitting errors. (**d**) Schematic illustration of the spatial organization of nascent DNA, RPA, and MCM within a replication fork derived from the Triple Correlation analysis.

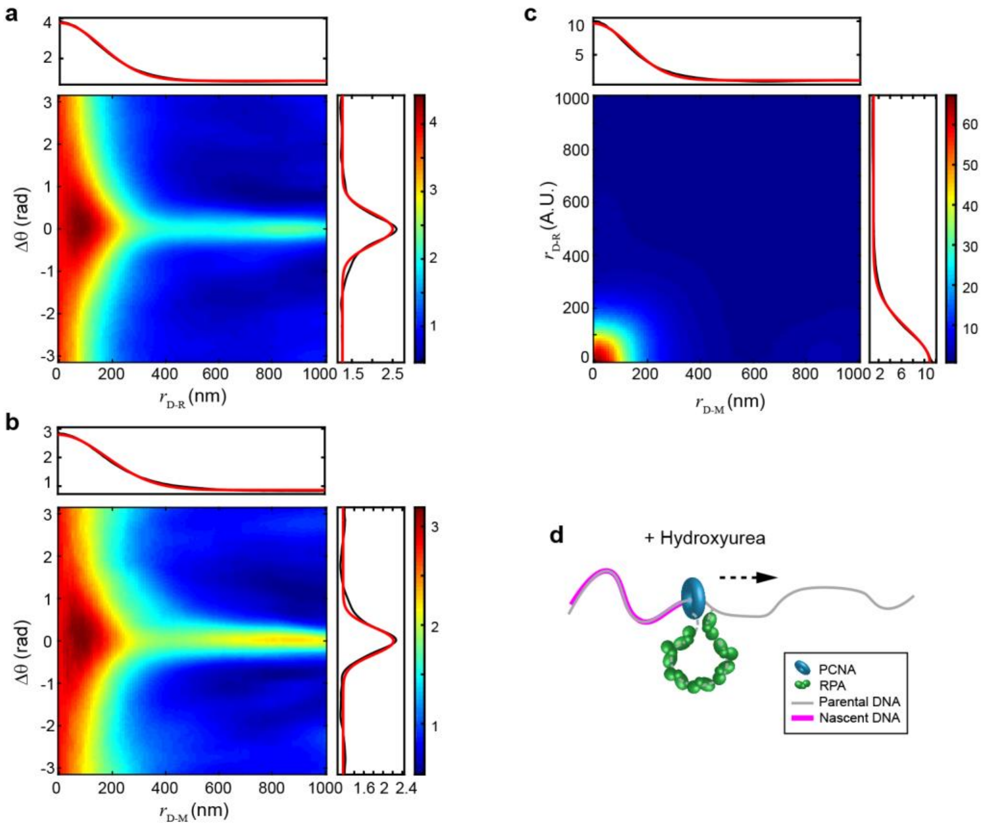

Supplementary Figure S9. Spatial organization of nascent DNA, PCNA, and RPA within replication fork foci in U2OS cells treated with 1 mM HU. (**a**) A 2D Triple-Correlation map as a function of the distance between DNA and RPA (r_{D-R}) and $\Delta\theta$, and its 1D correlation $g_{r_{D-R}}(r)$ (i, black line) and $g_{\Delta\theta}(\Delta\theta)$ (ii, black line), with the latter yielding a Gaussian distribution of $\Delta\theta$ centered at 0.00 with $\sigma = 21.2$ deg. (**b**) A 2D Triple-Correlation map as a function of the distance between DNA and PCNA (r_{D-P}) and $\Delta\theta$, and its 1D correlation $g_{r_{D-P}}(r)$ (i, black line) and $g_{\Delta\theta}(\Delta\theta)$ (ii, black lines), with the latter yielding a Gaussian distribution of $\Delta\theta$ centered at 0.00 with $\sigma = 18.3$ deg. (**c**) A 2D Triple-Correlation map as a function of r_{D-R} and r_{D-P}, and its 1D correlation $g_{r_{D-R}}(r)$ (i, black line) and $g_{r_{D-P}}(r)$ (ii, black lines). Their fits (red lines) yield correlation distances of $r_{D-R} = 100.7 \pm 0.6$ nm (with $r_0 = 12 \pm 8$, $\sigma = 125.8 \pm 0.8$, Equation 4), and $r_{D-M} = 109.8 \pm 0.9$ nm (with $r_0 = 12 \pm 11$, $\sigma = 137.2 \pm 1.0$, Equation 4). The Triple-Correlation is an average of Triple-Correlation profiles from 18 nuclei in U2OS cells. Errors are given as propagated fitting errors. (**d**) Schematic illustration of a replication fork that stalled due to depletion of dNTP pools, as derived from Triple Correlation analysis. Dashed arrow denotes replisome processing DNA replication at a slower speed.
